# Microbiota Dysbiosis a Cause of Colorectal Cancer or Not? A Systematic Review

**DOI:** 10.7759/cureus.30893

**Published:** 2022-10-31

**Authors:** Godfrey Tabowei, Greeshma N Gaddipati, Maria Mukhtar, Mohammed J Alzubaidee, Raga Sruthi Dwarampudi, Sheena Mathew, Sumahitha Bichenapally, Vahe Khachatryan, Asmaa Muazzam, Chandani Hamal, Lakshmi Sai Deepak Reddy Velugoti, Lubna Mohammed

**Affiliations:** 1 Department of Internal Medicine, California Institute of Behavioral Neurosciences & Psychology, Fairfield, USA; 2 Department of Research, California Institute of Behavioral Neurosciences & Psychology, Fairfield, USA; 3 Department of Pathology Research, California Institute of Behavioral Neurosciences & Psychology, Fairfield, USA; 4 Department of Internal Medicine/Department of Family Medicine, California Institute of Behavioral Neurosciences & Psychology, Fairfield, USA

**Keywords:** microbes or ("microbiota" [all fields] or "microbiome" [all fields]) and ("colorectal cancer" [all fields] or "colorectal carcinogenesis" [all fields] or "colon cancer" [all fields] or "rectal cancer" [all fields]), microbes associated with crc, colerectal carcinogenesis, microbial dysbiosis, microbes and colorectal cancer

## Abstract

Deaths from colorectal cancer (CRC) are still rising, and various links to etiology have been proposed. However, a direct link between microbial dysbiosis and colorectal cancer has not been postulated. This study aimed to identify the role of microbes in the pathogenesis of colorectal cancer. This systematic review was based on the Preferred Reporting Items for Systematic Reviews and Meta-Analyses (PRISMA) guidelines. A systematic search was done considering papers published over the past 12 years, using PubMed, PubMed Central, Cochrane, Google Scholar, and ScienceDirect databases. Studies were selected based on the following predefined eligibility criteria: English-language systematic reviews, meta-analysis, randomized controlled trials (RCTs), and clinical trials, which included papers on microbes playing roles in colorectal cancer with the derived data transferred to a template. Following this, quality assessment was done using each study's relevant assessment tool. The initial search generated 128 studies. From the study, we found the ratio of *Fusobacterium*, when compared between healthy and colorectal cancer patients' guts, was the highest, although it was not the most predominant gut organism. Enterotoxigenic *Bacteroides fragilis* (ETBF), *Clostridium* and* Salmonella*, and *Peptostreptococcus* showed links with colorectal cancer and described pathways that could explain its implication in colorectal cancer. However, overt conclusions cannot be drawn because further research needs to be conducted.

## Introduction and background

Colorectal cancer (CRC), also known as bowel cancer, colon cancer, or rectal cancer, is the development of cancer from the colon or rectum (parts of the large intestine). Signs and symptoms may include blood in the stool, a change in bowel movements, weight loss, and fatigue [1]. It is one of the most common cancers among men and women worldwide and has been noted to be the third most common cause of death from cancer and the fourth most common cause of cancer-related death, an incident of about 1.2 million new cases being reported every year around the world [2].

As with many diseases, the etiology of CRC is multifactorial, involving genetic and environmental factors. A study conducted on twins and family members showed that only a minute percentage of CRCs, including familial adenomatous polyposis (FAP), hereditary nonpolyposis colorectal cancer (HNPCC or Lynch syndrome), Peutz-Jeghers syndrome, and other uncommon forms of CRC, are genetically predisposed. To add to the above findings, most CRCs were sporadic and non-hereditary. Ecological issues, such as the Western diet, obesity, and hazardous alcohol intake, have been implicated as significant factors in sporadic CRCs. The intestinal microbiota has been noted to be a significant factor among the sporadic factors contributing to CRC, with new evidence revealing increased implications of these agents in the beginning, advancement, and spread of CRC to other parts of the body [3]. The human microbiota consists of organisms (bacteria, archaea, lower and higher eukaryotes, and viruses) found in and on the human body or their collective genomes (i.e., genetic material) [4].

A model gut usually contains trillions of microorganisms from several hundreds of distinct species, whose genetic material can contain over three million genetic codes worldwide [5]. The human intestine microbiota is colonized by three primary phyla, *Firmicutes* (30-50%), *Bacteroides* (20-40%), and *Actinobacteria*. Obligate anaerobes such as *Bifidobacterium, Fusobacterium, Bacteroides, Eubacterium, Peptostreptococcus*, and *Atopobium* are the dominant groups of bacteria in the gut. In contrast, facultative anaerobes such as *Lactobacilli, Enterococci,* and *Enterobacteriaceae* are usually 1,000-fold lower than obligate anaerobes [6].

The process of CRC starts with metaplasia of the normal gut epithelium, associated with increased proliferation. These metaplastic cells have abnormal function and composition, thereby predisposing them to adenoma formation. These adenomas can then increase in size and become malignant cells that can spread to the deeper layers of the gut and other body parts. This series of events, called the "adenoma-carcinoma sequence," which leads to CRC, is diversified, and various isoforms have been described depending on the genetic modifications. Three significant pathways have been implicated in the pathogenesis of sporadic CRC, and they include chromosomal instability (CIN), microsatellite instability (MSI), and cytosine-phosphate-Guanine Island methylator phenotype (CpGIMP). These pathways commutatively lead to tumor development and progression [7].

Consequently, the theorem which implicates microbes as crucial role players in the pathogenesis of CRC has been increasingly noticed; these agents cause modifications in the porosity of the gut mucosal lining, bacterial translocation, and trigger the immune system resulting in long-standing inflammation that could lead to CRC development [8]. CRC is usually diagnosed following the presence of occult blood in stool, which is generally followed by colonoscopy and biopsy for histology [2]. There is solid proof to show that ecological factors influence gut microbes; however, little is known about the direct link between microbial dysbiosis and CRC evolution [9]. This systematic review aims to review any direct links between microbes and CRC.

## Review

Methods

This systematic review was conducted based on the Preferred Reporting Items for Systematic Reviews and Meta-Analyses (PRISMA) 2020 guidelines [10].

Eligibility Criteria

The following inclusion criteria were used: Studies published from 2000-2022, the article should be written in English, and studies relevant to the subject (presenting original data). Free full texts, meta-analyses, randomized clinical trials (RCTs), and clinical trials must be in English. Studies unrelated to the topic, no access to full-text articles, case reports, reviews, animal studies, or books. Duplicated articles were excluded.

Selection Strategy

Two reviewers selected the articles independently using the same search strategy in all three journals. At first, articles were screened from the title of articles and abstracts and then later by reading full-text articles. If contradicting results regarding the article’s eligibility occurred, reviewers assessed the full-text article until the group reached a consensus.

Databases and Search Strategy

We searched electronic medical databases: PubMed, Google Scholar, and Science Direct, from January 2000 to January 2022 for all English human and non-human studies assessing the role of microbes in the pathogenesis of colorectal cancer. Keywords used in PubMed: "Microbes OR ("microbiota" [All Fields] OR "microbiome" [All Fields]) AND ("colorectal cancer" [All Fields] OR "colorectal carcinogenesis" [All Fields] OR "colon cancer" [All Fields] OR "rectal cancer" [All Fields])". Also, the use of Keywords such as Microbes and Colorectal Cancer was employed in getting data from Google Scholar and Science Direct.

All references were grouped and alphabetized using Microsoft Excel 2021 for duplicate removal. The records were initially reviewed based on the titles and abstracts, excluding irrelevant studies. Following review, a retrieval of the full-text articles was done. 

Risk of Bias in Individual Studies

The full articles remaining were assessed for quality assessment and risk of bias using tools depending on the study type; Cohort Studies, Newcastle Ottawa Scale (NOS) [11]; Systematic reviews and Meta-analyses, Assessment of Multiple Systematic Reviews 2 (PRISMA 2020 Checklist) [10]; and Narrative reviews, Scale for the Assessment of Narrative Review Articles (SANRA) [12]. Each assessment tool had its criteria and different scoring. A point is given when a tool scores "LOW RISK," "YES," and "PARTIAL YES," or "1". Two points are given when "2" is indicated. A score of at least 70% for each assessment tool was accepted. Table 1 summarizes the questions used to assess the qualities of the papers included in the study.

**Table 1:** Risk of bias using the various quality checklist PRISMA- Preferred Reporting Items for Systematic Reviews and Meta-Analyses; SANRA- Scale for the Assessment of Narrative Review Articles

Quality Assessment Tool	Type Of Study	Items and Their Characteristics	Total Score	Accepted Score (>70%)	Accepted Studies
PRISMA [10]	Systematic Review and Meta-analysis	Thirty-Four Items:1) Did the review authors Identify the report as a systematic review? 2) Did the review authors See the PRISMA 2020 for Abstracts checklist?3) Did the review authors describe the rationale for the review in the context of existing knowledge? 4)Did the review authors provide an explicit statement of the objective(s) or question(s) the review addresses? 5)Did the review authors specify the inclusion and exclusion criteria for the review and how studies were grouped for the syntheses? 6) Did the review authors specify all databases, registers, websites, organizations, reference lists, and other sources searched or consulted to identify studies? Specify the date when each source was last searched or consulted. 7)Did the review authors present the full search strategies for all databases, registers, and websites, including any filters and limits used? 8) Did the review authors specify the methods used to decide whether a study met the inclusion criteria of the review, including how many reviewers screened each record and each report retrieved, whether they worked independently, and if applicable, details of automation tools used in the process? 9)Did the review authors specify the methods used to collect data from reports, including how many reviewers collected data from each report, whether they worked independently, any processes for obtaining or confirming data from study investigators, and if applicable, details of automation tools used in the process? 10a) Did the review authors list and define all outcomes for which data were sought? Specify whether all results that were compatible with each outcome domain in each study were sought (e.g., for all measures, time points, analyses), and if not, the methods used to decide which results to collect. 10b) Did the review authors list and define all other variables for which data were sought (e.g., participant and intervention characteristics, funding sources)? Describe any assumptions made about any missing or unclear information. 11) Did the review authors specify the methods used to assess risk of bias in the included studies, including details of the tool(s) used, how many reviewers assessed each study and whether they worked independently, and if applicable, details of automation tools used in the process? 12) Did the review authors specify for each outcome the effect measure(s) (e.g., risk ratio, mean difference) used in the synthesis or presentation of results? 13a) Did the review authors describe the processes used to decide which studies were eligible for each synthesis (e.g., tabulating the study intervention characteristics and comparing against the planned groups for each synthesis? 13b) Did the review authors describe any methods required to prepare the data for presentation or synthesis, such as handling of missing summary statistics, or data conversions? 13c) Did the review authors describe any methods used to tabulate or visually display results of individual studies and syntheses? 13d) Did the review authors describe any methods used to synthesize results and provide a rationale for the choice(s)? If meta-analysis was performed, describe the model(s), method(s) to identify the presence and extent of statistical heterogeneity, and software package(s) used. 13e) Did the review authors describe any methods used to explore possible causes of heterogeneity among study results (e.g., subgroup analysis, meta-regression)? 13f) Did the review authors describe any sensitivity analyses conducted to assess the robustness of the synthesized results? 14) Did the review authors describe any methods used to assess the risk of bias due to missing results in a synthesis (arising from reporting biases)? 15) Did the review authors describe any methods used to assess certainty (or confidence) in the body of evidence for an outcome? 16a) Did the review authors describe the search and selection process results, from the number of records identified in the search to the number of studies included in the review, ideally using a flow diagram? 16b) Did the review authors cite studies that might appear to meet the inclusion criteria but which were excluded, and explain why they were excluded? 17)Did the review authors cite each included study and present its characteristics? 18) Did the review authors present bias risk assessments for each included study? 19) Did the review authors for all outcomes present for each study: (a) summary statistics for each group (where appropriate) and (b) an effect estimate and it's precision (e.g., confidence/credible interval), ideally using structured tables or plots? 20a) Did the review authors for each synthesis briefly summarise the characteristics and risk of bias among contributing studies? 20b) Did the review authors present the results of all statistical syntheses conducted? If meta-analysis was done, present for each the summary estimate and its precision (e.g., confidence/credible interval) and measures of statistical heterogeneity. If comparing groups, describe the direction of the effect. 20c) Did the review authors present results of all investigations of possible causes of heterogeneity among study results? 20d) Did the review authors present the results of all sensitivity analyses conducted to assess the robustness of the synthesized results? 21) Did the review authors present assessments of risk of bias due to missing results (arising from reporting biases) for each synthesis assessed? 22) Did the review authors present certainty (or confidence) assessments in the body of evidence for each outcome assessed? 23a) Did the review authors provide a general interpretation of the results in the context of other evidence? 23b) Did the review authors discuss any limitations of the evidence included in the review? 23c) Did the review authors discuss any limitations of the review processes used? 23d) Did the review authors discuss the implications of the results for practice, policy, and future research? 24a) Did the review authors provide registration information for the review, including register name and registration number, or state that the review was not registered? 24b) Did the review authors Indicate where the review protocol can be accessed or state that a protocol was not prepared? 24c) Did the review authors describe and explain any amendments to the information provided at registration or in the protocol? 25) Did the review authors describe sources of financial or non-financial support for the review and the role of the funders or sponsors in the review? 26) Did the review authors declare any competing interests of review authors? 27) Did the review authors report which of the following are publicly available and where they can be found template data collection forms; data extracted from included studies; data used for all analyses; analytic code; any other materials used in the review? Scored as 0,1.	44	31	Vandenbulcke et al. 2020 [4] Borges-Canha et al. 2015 [13] Reitano et al. 2021 [14] Scott et al. 2022 [15]
SANRA [12]	Narrative Review	Six items: justification of the article’s importance to the readership, statement of concrete aims or formulation of questions, description of the literature search, referencing, scientific reason, and appropriate presentation of data. Scored as 0, 1 or 2.	12	9	Gao et al. 2015 [9] Ye et al. 2017 [16] Tahara et al. 2014 [17] Castellarin et al. 2012 [18]
New Castle Ottawa [11]	Cohort	Eight items: (1) Representativeness of the exposed cohort (2) Selection of the non-exposed cohort (3) Ascertainment of exposure (4) Demonstration that outcome of interest was not present at the start of study (5) Comparability of cohorts based on the design or analysis* (6). Assessment of outcome (7) Was follow-up long enough for outcomes to occur (8) Adequacy of follow-up of cohorts Scoring was done by placing a point on each category. Scored as 0, 1, 2. * Maximum of two points are allotted in this category.	8	6	Tsoi et al. 2017 [19]

Table 2 summarizes the characteristics of the papers included in the study.

**Table 2:** Characteristics of papers included in the study rRNA- Ribosomal ribonucleic acid; CRC-Colorectal Cancer; DNA-Deoxy-ribonucleic acid; PCR- Polymerase Chain reaction; qPCR- Quantitative Polymerase Chain reaction; ETBF-Enterotoxigenic *Bacteriodes fragilis*; RNA-seq-Ribosomal nucleic acid sequence; GA11x- Genome Analyzer 11x; *F.nucleatum*- *Fusobacterium nucleatum*; CpG islands- Cytosine-Phosphate-Guanine Islands; rDNA-Ribosomal Deoxy-ribonucleic acid; IL17A- Interleukin 17A; 1L21- Interleukin 21; TNF- Tumour necrosis factor; CCL20- Chemokine ligand 20; *P.anaerobius*-* Peptostreptooccus anaerobius*; ROS- Reactive Oxygen Species; TLR2-Toll-like receptor 2; TLR4- Toll-like receptor 4

Author	Year Of Study	Type Of Study	Methods	Limitations	Conclusion
Vandenbulcke et al. [4]	2020	Systematic Review	Next-generation sequencing approaches, including 16S rRNA gene amplicons and genome shotgun metagenomics -aerobic and anaerobic subculturing onto selective agar media	It was a problem to obtain clear and quality evidence that different organisms might play a role in the various steps of CRC pathogenesis.	However, there was a strong link between specific microbes and CRC; it is not entirely possible to have a definitive conclusion on a microbe's CRC risk since the data was mixed, and merging studies for meta-analysis is extremely difficult.
Reitano et al. [14]	2021	Systematic Review	DNA analysis, with 16S rRNA V4 sequencing and PCR - indirect immunofluorescence -shotgun sequence	The quality of data used in the study	Irrespective of the few available data, the study shows a significant association between microbes and CRC cancer. However, it is not easy to pick out a single organism in the development of CRC. Further studies are needed to characterize these organisms.
Borges-Canha et al. [13]	2015	Systematic Review	Biopsy from fecal samples of humans and animals (mice)	Different methodologies used by different authors might lead to errors in the results obtained. Human and animal studies were both used in the study, with equal importance given to them.	Despite evidence linking microbial dysbiosis to CRC, it is impossible to firmly conclude that dysbiosis is a cause or a consequence of CRC; future research in this area is needed.
Scott et al. [15]	2022	Systematic Review	qPCR for detection meta transcriptomic data to search for bacterial toxin gene expression	The Heterogenicity of the studies used is a potential for bias.	Since the studies have brought to light an essential relationship between ETBF and CRC, a combination of high-quality research would be necessary to explain this hypothesis further.
Castellarin et al. [18]	2012	Narrative review	Biospecimens were held briefly at −20°C during frozen sectioning, using 100% ethanol -Illumina RNA-seq libraries were constructed, barcoded, and pooled, and two lanes of paired-end sequencing data were obtained using the Illumina GAIIx platform.		Our observation of a highly significant over-representation of *F.nucleatum* in colorectal tumor specimens was largely unexpected, given that it is generally regarded as an oral pathogen—it is not an abundant constituent of the normal gut microbiota. This supports the notion that a comprehensive study of early-stage lesions may help determine whether *Fusobacterium* infection is related to the early stages of tumor progression.
Gao et al. [10]	2015	Narrative Review	Pyrosequencing-based analysis of 16S rRNA genes		The study suggests gut dysbiosis is associated with CRC risk primarily through metabolic exchange or direct interaction with the host.
Tahara et al. [17]	2014	Narrative Review	Genomic DNA samples Quantitative real-time PCR		Patients with a high level of *Fusobacterium* in their CRC tissues have a molecularly distinct type of cancer, with a high degree of CpG island methylation and a high rate of mutations. These data implicate *Fusobacterium* as a virulent factor in CRC pathogenesis rather than a mere passenger.
Ye et al. [16]	2017	Narrative Review	Illumina next-generation analysis of bacterial 16S rDNA in the frozen tissue specimens -normoxic culture was performed in an incubator using MycoAlert mycoplasma detection kit and AnaeroGen Compact system		CRCs differentially expressed the proinflammatory cytokines IL17A, IL21, TNF, and CCL20, with CCL20 being highly expressed at every stage of CRC. In *in vitro* assays, coculture with *F.nucleatum* species (*Animalis)* markedly induced CCL20 protein expression in specific colorectal cancer cells, which implicates it in CRC development.
Tsoi et al. [19]	2017	Cohort	Analyses stool and mucosa samples by metagenomics four and 16S ribosomal RNA gene sequencing.24 -qPCR		*P.anaerobius* is associated with CRC and could be one of the potential driver's bacteria in tumor development*. P.anaerobius *promotes cholesterol synthesis and CRC cell proliferation by enhancing ROS production through interaction with TLR2 and TLR4.

Results

**Figure 1:**
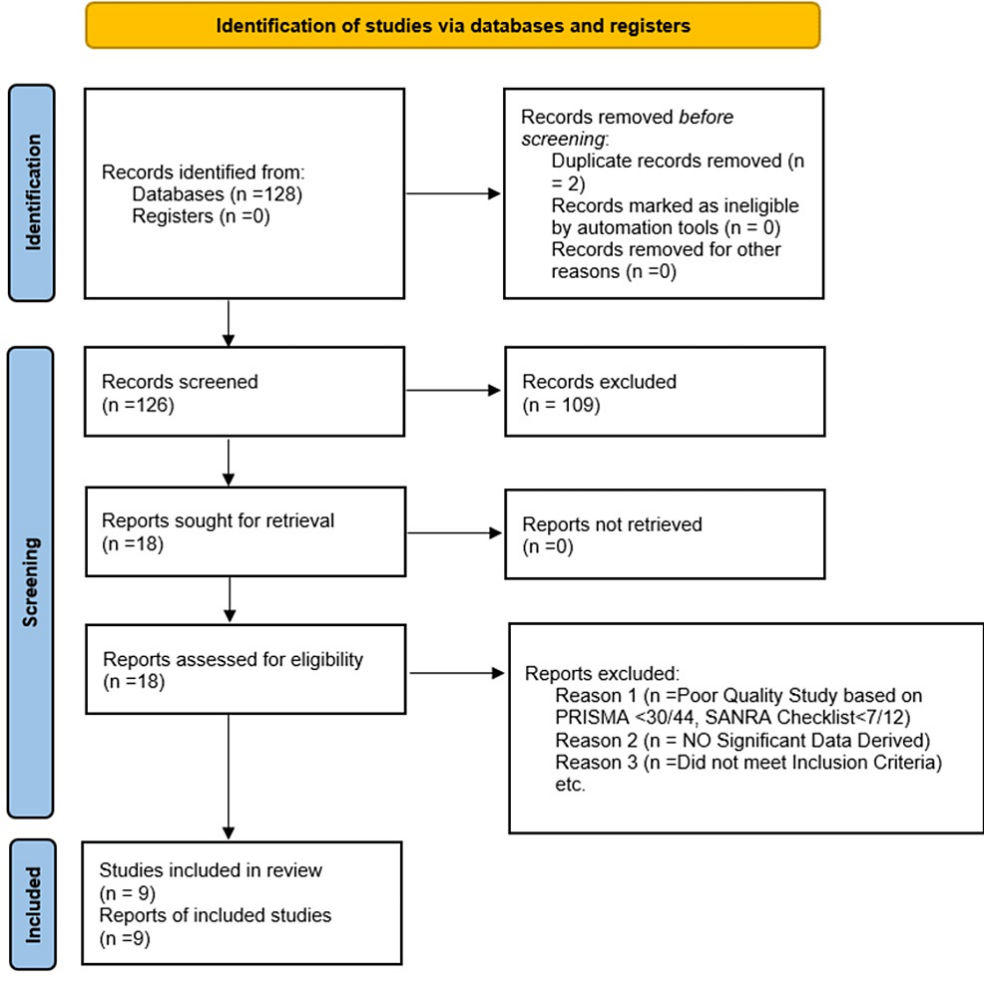
Flow chart of the study selection process PRISMA- Preferred Reporting Items for Systematic Reviews and Meta-Analysis; SANRA- Scale for Assessment of Narrative Review Articles.

The database search found 128 potentially related titles based on the study inclusion criteria. 126 records were kept after duplicates were removed. When the titles and abstracts were evaluated in detail, 18 articles remained. Nine papers with a score of 70 percent and above were allowed in the review after the 18 publications underwent a quality assessment screening. There were four systematic reviews, one RCT, and four narrative reviews. A flow diagram of the study selection and screening process is shown in Figure 1 [10].

Discussion

**Figure 2:**
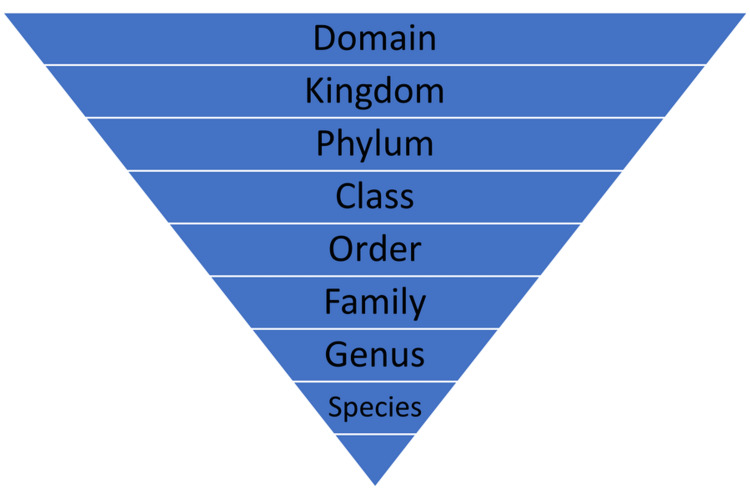
Hierarchal classification of organisms Created by the authors using Microsoft Word

From ongoing research, there are proposed links through which microbiome dysbiosis has been proposed and linked to causing CRC. This review will highlight these links by identifying different alterations in gut microorganisms, establishing the pathogenic mechanisms via which organisms induce immune signaling and inflammation, and then showing specifics of some organisms linked to CRC [13]. A pictorial diagram created using Microsoft Word representing the hierarchal classification of organisms is shown in Figure 2.

Alterations in Gut Microbiome

A study showed notable microbial differences in the gut of colorectal cancer patients and healthy patients; bacteria from the gut of healthy individuals and individuals with colorectal cancer were examined. The results following the survey showed that *Firmicutes* accounted for about 63.46% of the microbial population (the chief organisms encountered) in colorectal cancer patients. In comparison, it accounted for 43.46% of healthy individuals% (95% Confidence Interval (CI): 5.67-34.32%, probability (P): < 0.001). In contrast,* Proteobacteria *were the most predominant organisms demonstrated in the gut of well persons, with a summing up to a percentage of 60.35, as compared to that seen in colorectal cancer, which was only present in small numbers of 10.66% (95% CI: −35.32 to −15.57%, P: < 0.001) [9]. Another study supports these findings in colorectal adenoma patients and healthy individuals as controls, which also showed a predominant amount of *Proteobacteria* in colorectal adenoma patients and a lower amount of *Bacteroides* in both colorectal adenoma patients and healthy individuals [8].

*Bacteroidetes* were the second most encountered group of organisms in both groups, CRC (12.7%) and Healthy individuals (13%), with 95% CI: −4.99 to 5.44%, P: > 0.05, respectively (P > 0.05), followed by* Fusobacteria* which was the third most abundant organism seen in the gut of both CRC (10.58%) compared with 0.03% in healthy humans (95% CI: 0.25-12.68%, P: < 0.001). A notable difference was that *Peptostreptococcusten species* were enriched in CRC patients. However, *Epilithonimonas, Flavobacterium (Flavobacteria), Pedobacter, Sphingobacterium (Sphingobacteria), Caulobacter, Brevundimonas, Sphingomonas (Alphaproteobacteria), Acidovorax, Janthinobacterium (Betaproteo- bacteria), Buttiauxella, Rahnella, Acinetobacter, Janthinobacterium, Psychrobacter, Pseudomonas, Stenotrophomonas (Gammaproteobacteria), Psychrobacter, Propionic- bacterium (Actinobacteria*) were reduced in CRC patients [9].

Depletion of Bacterial Communities in Adenoma 

One of the studies conducted showed that there was a decrease in some organisms in patients with colorectal cancer/adenoma in comparison to the total amount in health patients; these differences were observed via a high-throughout 454 pyrosequencing of human feces obtained from forty-seven patients matched by similar characteristics (age, sex, and lifestyles). The fecal sample derived from colorectal adenoma patients revealed differences in their constituents when matched with those of healthy individuals. *Clostridium, Roseburia, and Eubacterium species*, in addition to the genera that metabolize butyrate, were noted to be depleted; surprisingly, Enterococcus, Streptococcus species, and Proteobacteria phylum were abundant when juxtaposed with the healthy patient's group [8]. 

In another study conducted in 2018 on twelve patients, similar inferences were made following analysis of biopsies derived from margins of healthy tissues and colonic polyps. Organisms from the group of the *Actinobacteria* phylum, such as the *Bifidobacterium *genus, were increased in the healthy mucosal tissue than in the polyp sample. Interestingly, noticed to be decreased were *Faecalibacterium, Bacteroides,* and *Romboutsia* [20].

Immune Signaling and Inflammation

The dysbiosis of microbiota in the gut leads to changes in immunologic signaling and, subsequently, chronic inflammation resulting in the pathogenesis of colorectal cancer. Interleukin-17 immunoreactive cells (mostly cluster of differentiation 3) were higher than average on pathologic and healthy mucosa analysis in colorectal cancer patients. An incidental finding was that not only interleukin-17A was above average, but interleukin-17C, which is a significant cytokine in the signaling pathway of colorectal cancer, promotes development by activating anti-apoptosis markers- B-cell lymphoma (Bcl-2) and B-cell lymphoma- extra-large (Bcl-xL) was also noticed to be increased in colorectal cancer patients when studied in both humans and animal model (mouse) [13].

Another study conducted also had similar findings of increased interleukin-17A in colorectal cancer patients; additionally, they also found an increase in the levels of tissue necrotic factor (TNF) and chemokine ligand 20 (CCL20) protein in colorectal cancer tissue when compared to adjacent normal healthy tissue. The role of chemokine ligand 20 and Chemokine receptor type 6 (CCR6), its receptor is notable in the pathogenesis of colorectal cancer, enlisting the body's defense cells, and has a contradictory role in regulating inflammation and immune tolerance [16]. An animal study conducted in mice has also shown overexpression of chemokine ligand 20, and its receptor chemokine receptor type 6 is incredibly significant in the pathogenesis of colorectal cancer [21].

A study showed that cell surface epithelial cadherin (E-cadherin) was the primary target of fragilysin, a significant constituent of the cell-to-cell binding structure (zonula adherens). E-cadherin is broken down in the presence of Fragilysin, leading to this protein's complete dissolution. Loss of this E-cadherin cell-to-cell binding property leads to a series of cascades. It triggers the movement of Beta-catenin (B-catenin) to the nucleus, binds to T-cell factor-dependent transcriptional activators, and activates c-myelocytomatosis oncogene product (c-Myc) transcription and translation, resulting in uncontrolled cellular proliferation [22]. Signal transducer and activator of transcription (STAT) proteins, especially signal transducer and activator of transcription protein 3 (STAT-3), are crucial for cancer inflammation's extrinsic and intrinsic pathways. Interleukin-6 signals are partially mediated by the activation of signal transducer and activator of transcription protein 3, a transcription factor important in cancer development [23].

Clostridium species

An animal study reveals that gut microbes provide substrates that lead to colorectal cancer formation through a pathway that is different from common ones. Findings in the study demonstrated that the etiology of colorectal cancer occurs following the administration of butyrate compound to Adenomatous polyposis coli-Multiple intestinal neoplasia/mismatch repair protein 2 (APCMin/+ MSH2-/-) mice, which leads to a polyp formation. Although butyrate has been formerly known to have anticancer characteristics through its action as histone deacetylase inhibitor (HDACi), when its levels are suboptimal, it leads to colonic epithelial cell replication, these discrepancies in the role of butyrate are called the "butyrate paradox." *Firmicutes* which include *Clostridium*, are the primary organisms that generate butyrate. *Clostridium* was implicated in the APCMin/+ MSH2-/- mice disease pathology. It was proposed that gut microbes play a role in the pathogenesis of colorectal cancer by providing substrates that promote the proliferation of epithelial cells [24]. 

Bacteroides species

In twenty-six studies carried out where mice were either given enterotoxigenic *Bacteroides fragilis* toxin or non-toxigenic *Bacteroides fragilis* (NTBF), 24 out the 26 revealed pathogenic induced traits that were linked with CRC, and the 26 studies that colonized mice with enterotoxigenic *Bacteroides fragilis* toxin and non-toxigenic *Bacteriodes fragilis*, comprised tumor formation in 11 of the studies; other findings notes included gut mucosal inflammation shown in six studies, polyp formation in two studies, colitis in six studies; other findings reported were ulcers, splenic enlargement, and macroadenoma [15]. Amongst the Bacteroides spp, there was a notable rise in the amount of *Bacteroides massileinses* and *Bacteroides dorei* from healthy patients to patients with colonic adenoma. This inference was derived after evaluating 156 metagenomic shotgun-sequenced fecal samples. Following the experiment, a significant correlation was noted between *Bacteroides dorei* and C-reactive protein, an acute phase reactant that plays a role in acute inflammation, thereby predisposing adenoma formation. The differences in the gut composition in healthy and colorectal cancer patients were also studied. A progressive rise in the diversification of genes was noted, with the rise increasing starting from the control and peaking in colorectal cancer patients. This infers that patients with high-stage colorectal cancer have more gene mutations, showing overgrowth of various harmful organisms. The authors also studied the varieties and uniformity of the gut microbiota in healthy controls compared to patients with advanced adenoma or carcinoma. A progressive increase in the genetic variation was highlighted, with a progressive increase starting from the controls to reaching the top in the carcinoma patients. Thus, in patients with advanced colorectal adenoma or carcinoma, many genes or genera likely indicate an overgrowth of harmful bacteria or archaea [8].

In another study, Zinc-dependent metalloprotease toxin was secreted by enterotoxigenic *Bacteriodes fragilis*, called *Bacteroides fragils* toxin (BFT), which breaks down E-cadherin resulting in the translocation of beta-catenin to the nucleus, then ultimately results in upregulation of c-Myc expression and cellular proliferation-colorectal cancer [25]. This was further buttressed in another study which proposed that Enterotoxigenic *Bacteroides fragilis *is a driver organism in colorectal cancer. It was hypothesized that *Bacteroides fragilis *toxin was enormously increased in colorectal adenoma patient's stool samples when compared with that of healthy patient's stool samples; the result from this study also supported this and commented that *Bacteroides fragilis* toxin activates the Wingless/Integrated (Wnt) pathway, which led to uncontrolled cellular proliferation after altering the E-cadherin/B-catenin interactions [26].

Fusobacterium species

Five species of *Fusobacterium* have been identified in colorectal cancer, with *Fusobacterium nucleatum* being the most predominant subspecies. Analysis of colorectal cancer showed that the tumor cells highly expressed proinflammatory cytokines, interleukin-7A, interleukin-21, tumor necrotic factor, and chemokine ligand 20 [16]. An animal study showed an acceleration in the development of colorectal cancer in the APCMin/+ mouse model following the administration of human isolates of* Fusobacterium nucleatum* compared to mice fed with *Streptococcus species* (p < 0.001) [13]. 

Products of *Fusobacterium species* were found in increased amounts in colorectal cancer. This conclusion was obtained from quantitative polymerase chain reaction and sequence analysis of 16S ribosomal deoxyribonucleic acid (16S rDNA) performed on 95 pairs of deoxyribonucleic acids (healthy and diseased) and by fluorescence in situ hybridization (FISH). Various mechanisms postulated that *Fusobacteria* gives rise to colorectal cancer, including pattern recognition receptors and inflammation leading to the enlistment of myeloid cells to infiltrate adenomas and carcinomas, resulting in Toll-like receptor 4 (TLR4) and nuclear factor kappa B (NF-kB) dependent signaling. *Fusobacterium* has also been associated with an increased release of inflammatory mediators, which include interleukin-1B, interleukin-6, and interleukin-8, possibly via the activation of TLR2/TLR4 [13]. The toll-like receptors are a significant receptor for lipopolysaccharide; when activated, it leads to a series of reactions that ultimately lead to the expression of interleukin-8. *Fusobacterium nucleatum* has not been known to be associated with any toxins; however, it encodes some pathogenic factors, which include *Fusobacterium nucleatum* adhesin A (FadA). The presence of *Fusobacterium nucleatum* adhesin a facilitates *Fusobacterium nucleatum* entry and attachment as a bacteria into the gut. *Fusobacterium nucleatum* adhesin A pathogenesis is aided by following its binding to an E-cadherin rector, which promotes cancer formation. *Fusobacterium nucleatum* adhesin A also activates beta-catenin and stimulates the release of several transcription factors, Wnt genes, inflammatory genes, and oncogenes. The adhesion to cells is also proposed to be aided by MORN repeat-containing protein 2 (MORN2), but its exact role is unknown. *Fusobacterium nucleatum* also binds to Cadherin 1 gene (CDH1), which promotes carcinogenesis [2]. Similar findings were also seen in a study where Illumina's next-generation analysis of bacterial 16S rDNA in the frozen tissue specimens obtained from patients with colorectal cancer was done. In addition to the above, *Fusobacterium nucleatum* strongly induces chemokine ligand 20 protein expression and subsequently promotes colorectal cancer pathogenesis after interacting with monocytes and recruiting other inflammatory cells [16]. 

Peptostreptococcus species

*Peptostreptococcus anaerobius* is populated in the gut and stool samples of colorectal cancer patients by next-generation sequencing technology. It was concluded that *Peptostreptococcus anaerobius* colonization might put an individual at risk of developing colorectal cancer. The study provided more data on the possible pathway *Peptostreptococcus anaerobius* leads to colorectal cancer. It was shown to be mediated by the interaction of *Peptostreptoccus anaerobius* and toll-like receptor-2/4, which induces the formation of reactive oxygen species (ROS), and regulates sterol regulatory element-binding protein 2 (SREBP2), which increases fat formation, activates pro-oncogenic factors and pathways that predispose to colorectal cancer [19].

Salmonella species

A study aimed to assess the role of Avra bacterial protein in colorectal cancer development via the STAT-3 activating pathway. In the study, the role and mechanism of a chronic infection of *Salmonella typhimurium* were involved in the pathogenesis of colorectal cancer in an Azoxymethane/Dextran sodium sulfate (AOM/DSS) colorectal cancer model. Significant findings in the study were changes in the number of immune cells by the Salmonella infection and activation of the STAT-3 pathways. They increased proliferating cell nuclear antigen (PCNA), a marker of increased colonic cellular proliferation. Avra bacterial protein was noted to increase the activation of the STAT-3 pathway continuously, and other STAT-3 genes were noted to be involved in colorectal cancer pathogenesis. STAT-3 induces inflammation by increasing interleukin-6 levels [23]. From this study, multiple associations with these organisms and the pathways via which they are implicated in colorectal cancer were established. However broader studies would be needed to ensure these organisms are the sole virulent factors in colorectal cancer without the additive effects of cofactors that can predispose to colorectal cancer.

Limitation

This review was limited to studies only in the English language. Grey literature and non-free full texts were also excluded, which decreased the amount of data used in the study. The review was also restricted during the search strategy because only a few papers dealt with the broad topic of microbes' role in the pathogenesis of colorectal cancer, and most of the studies dealt with specific microorganisms. Some of the studies varied in their findings and used different methodologies, and most of the studies just proposed the mechanisms via which the organisms can cause colorectal cancer; However, some data derived were significant; conclusions as to whether to classify an organism as a direct cause of colorectal cancer was not obtained; just postulates and statements linking them together were made.

Furthermore, most of the studies that showed the organisms as causal factors for colorectal cancer were carried out in animals; human studies trials were not done. Therefore, this review recommends more in-depth RCTs and clinical trials to demonstrate direct causal factors to colorectal cancer after excluding other factors that can contribute to colorectal cancer; then, we can surely say that these organisms directly cause colorectal cancer. 

## Conclusions

The pathogenesis of colorectal cancer has been known to be multifactorial, cutting across genetic and environmental factors interplay. However, a direct link between microbes and colorectal cancer is still an unanswered question. From the review above, it was noted that there was microbial dysbiosis in a patient with colorectal cancer, predominantly *Fusobacterium,* and the population of these organisms was noted to be depleted in the gut of healthy individuals. The role of these organisms in the pathogenesis of colorectal cancer was not clearly stated; however, there were links explained via the activation of various signaling pathways and upregulation of some inflammatory cytokines, which lead to the transformation of normal gut epithelium to malignant cells. The organisms from the study which showed more association with colorectal cancer were *Fusobacteria *and enterotoxigenic* Bacteroides fragilis* toxin. Moving forward, it would be helpful if more animal and clinical studies could be done to isolate the organisms as the cause of colorectal cancer and see if eradication of these organisms can help prevent the disease.
